# Validation of a P-Glycoprotein (P-gp) Humanized Mouse Model by Integrating Selective Absolute Quantification of Human MDR1, Mouse Mdr1a and Mdr1b Protein Expressions with *In Vivo* Functional Analysis for Blood-Brain Barrier Transport

**DOI:** 10.1371/journal.pone.0118638

**Published:** 2015-05-01

**Authors:** Muhammad Waqas Sadiq, Yasuo Uchida, Yutaro Hoshi, Masanori Tachikawa, Tetsuya Terasaki, Margareta Hammarlund-Udenaes

**Affiliations:** 1 Department of Pharmaceutical Biosciences, Uppsala University, Uppsala, Sweden; 2 Division of Membrane Transport and Drug Targeting, Graduate School of Pharmaceutical Sciences, Tohoku University, 6–3 Aoba, Aramaki, Aoba-ku, Sendai, Japan; Hungarian Academy of Sciences, HUNGARY

## Abstract

It is essential to establish a useful validation method for newly generated humanized mouse models. The novel approach of combining our established species-specific protein quantification method combined with in vivo functional studies is evaluated to validate a humanized mouse model of P-gp/MDR1 efflux transporter. The P-gp substrates digoxin, verapamil and docetaxel were administered to male FVB Mdr1a/1b(+/+) (FVB WT), FVB Mdr1a/1b(-/-) (Mdr1a/1b(-/-)), C57BL/6 Mdr1a/1b(+/+) (C57BL/6 WT) and humanized C57BL (hMDR1) mice. Brain-to-plasma total concentration ratios (K_p_) were measured. Quantitative targeted absolute proteomic (QTAP) analysis was used to selectively quantify the protein expression levels of hMDR1, Mdr1a and Mdr1b in the isolated brain capillaries. The protein expressions of other transporters, receptors and claudin-5 were also quantified. The K_p_ for digoxin, verapamil, and docetaxel were 20, 30 and 4 times higher in the Mdr1a/1b(-/-) mice than in the FVB WT controls, as expected. The K_p_ for digoxin, verapamil and docetaxel were 2, 16 and 2-times higher in the hMDR1 compared to the C57BL/6 WT mice. The hMDR1 mice had 63- and 9.1-fold lower expressions of the hMDR1 and Mdr1a proteins than the corresponding expression of Mdr1a in C57BL/6 WT mice, respectively. The protein expression levels of other molecules were almost consistent between C57BL/6 WT and hMDR1 mice. The P-gp function at the BBB in the hMDR1 mice was smaller than that in WT mice due to lower protein expression levels of hMDR1 and Mdr1a. The combination of QTAP and in vivo functional analyses was successfully applied to validate the humanized animal model and evaluates its suitability for further studies.

## Introduction

The use of humanized mice models to study drug transport in vivo is on the rise with many promising models in the discovery pipeline. It is essential to establish a useful method to validate newly generated humanized mouse models including the MDR1 humanized mice. One of the most important issues is how we should distinguish the functionality of the introduced human gene from that of the mouse one which should ideally be completely replaced with the human. Although it is necessary to prove absence of expression and function of the mouse molecule in the humanized mice, it is quite difficult because the functionalities often overlap between mice and humans. An absolute quantification method has recently been established to selectively determine the protein expression levels of the targeted molecules based on the differences in amino acid sequences, thereby enabling us to distinctively quantify the protein expressions of mouse and human molecules[1]. Protein expression level is likely to correlate with the function compared to mRNA expression level.

Pharmaceuticals supposed to act in the central nervous system (CNS) are dependent on the passage through the BBB and entry in the brain. P-glycoprotein (P-gp) is the most important transport protein at the BBB [2], functioning as an efflux pump and limiting the brain uptake of many of these pharmaceuticals. P-gp was also the very first drug efflux transporter to be discovered and is the most extensively studied member of the ATP binding cassette (ABC) multidrug transporter family [3, 4]. Therefore the knowledge of different expression levels and functional dissimilarities in P-gp is important for correct correlation between animal experiments and clinical drug development. There is also a risk that a substance supposed to act within the CNS that proves to be a P-gp substrate during drug development and maybe gives low concentration in the brain in an animal model, is excluded and not further investigated. Potentially good pharmaceuticals can then be rejected on the wrong basis.

Despite its importance, there is still uncertainty concerning the existence of species differences and their significance [5, 6]. Thus, there is a requirement for more studies in this area, exploring the existence of species differences in the P-gp transport of pharmaceuticals to establish sound basis for correlation of preclinical studies to clinical research. Most of the knowledge we have about the effect of P-gp on the brain pharmacokinetics of different drugs is from in vivo experiments on rodents (rats and mice). However, rodents are not humans and clinical in vivo studies are limited by ethical and technical restrictions. In humans there is only one P-gp transporter encoded by the gene MDR1, while mice have two variants encoded by Mdr1a and Mdr1b performing the same function [7, 8]. Mice lacking the Mdr1a gene, were developed during the 90s and were a very important contribution to studies of the role of P-gp in vivo [9]. The Mdr1a(-/-) mice were 100-fold more sensitive to the neurotoxic pesticide ivermectin and 3-fold more sensitive to vinblastine, when compared to wild type controls, emphasizing the importance of P-gp at the BBB [9]. While Mdr1a expression in rodent BBB and its function as an efflux pump has been described over the last 20 years, the experimental proof of the functionality of MDR1 at the human BBB was found using imaging technology [10]. More recent studies indicate differences in the P-gp function and expression among different species. Species differences within rodents was indicated when Cutler and coworkers found that a higher blood concentration of the P-gp inhibitor elacridar (GF120918) was needed in guinea pigs to obtain the same inhibition as in rats and mice [11]. In another study, the brain penetration of [18F]altanserine and [11C]GR205171 in humans was 4.5 and 8.6 times higher than in rodents [6]. Even though imaging techniques are limited by the availability of specific substrates, these results suggest that P-gp is more active or expressed at higher abundance at the BBB in mice compared to humans. An MDR1 humanized mouse model (hMDR1) has been developed in which both the mouse *Mdr1a* and *Mdr1b* genes were intended to be replaced with human *MDR1*. This model is expected to allow us to conduct functional in vivo studies of human P-gp in mice, thus possibly bypassing some of the existing ethical and practical restrictions.

Therefore, the aim of the present study was to validate the MDR1 humanized mice model using a novel approach of integrating the in vivo functional analysis with quantitative targeted absolute proteomic (QTAP) analysis.

## Materials and Methods

### 2.1. Generation of the hMDR mouse model

#### 2.1.1. DNA constructs and cloning

**Fig 1:**
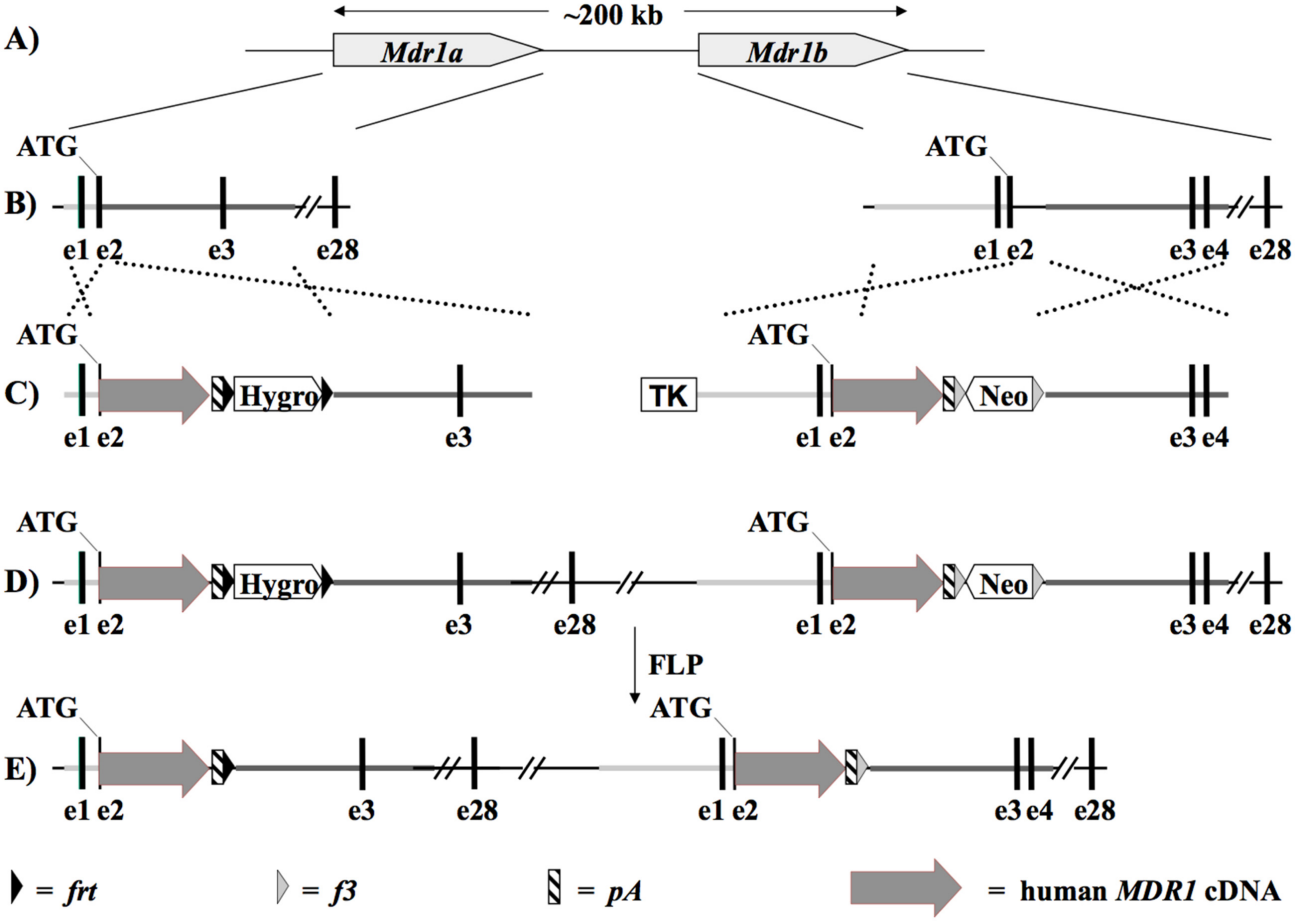
Stages for the development of P-gp hMDR1 mice described in different steps during which both *Mdr1a* and *Mdr1b* were knocked out from mouse DNA and replaced by human *MDR1*.

For targeting the *Mdr1a* gene locus a basic vector containing (1) a Hygromycin expression cassette flanked by *frt* sites, (2) a 1.1 kb genomic sequence upstream of the translational start ATG of the mouse *Mdr1a* gene on exon 2 and a 6.2 kb genomic sequence downstream of exon 2 used as targeting arms for homologous recombination and (3) a cDNA of human *MDR1* fused to the mouse translational start ATG and followed by a polyadenylation motif was constructed in pBluescript (pBS) as depicted in Fig 1. The human *MDR1* cDNA was generated by TaconicArtemis GmbH (Cologne, Germany) by subcloning of PCR fragments amplified from different human cDNA libraries. The human *MDR1* cDNA used for the final targeting vector was corrected by TaconicArtemis for polymorphic nucleotide changes, such that it codes for a protein that matches the human P-glycoprotein reference sequence (http://www.uniprot.org/uniprot/P08183).

For targeting the *Mdr1b* gene locus the same human *MDR1* cDNA was fused to the translational start ATG of mouse *Mdr1b*. In this case the basic targeting vector also contained a 5.2 kb genomic sequence upstream of the translational start ATG of the mouse *Mdr1b* gene on exon 2 and a 6.9 kb genomic sequence downstream of exon 2 used as targeting arms for homologous recombination, as well as a Neomycin expression cassette flanked by *f3* sites and a thymidine kinase expression cassette located outside of the targeting arms (Fig 1).

#### 2.1.2. Generation and molecular characterization of targeted embryonic stem cells

Culture and targeted mutagenesis of embryonic stem (ES cells) were carried out by TaconicArtemis as described previously [12]. *Mdr1a*: The targeting vector was linearized with *Not*I and electroporated into a C57BL/6 mouse ES cell line. Of 367 hygromycin resistant ES cell colonies screened by standard Southern blot analyses, six correctly targeted clones were identified, expanded and further analyzed by Southern blot analyses with 5’ and 3’ external probes and an internal hygromycin probe. All six clones were confirmed as correctly targeted at both homology arms without additional random integrations (data not shown). *Mdr1b*: The targeting vector was linearized using *Fse*I and electroporated into one of the correctly targeted *Mdr1a* ES clone described above. Of 662 G418 and gancyclovir resistant ES cell colonies screened by standard Southern blot analyses 13 correctly targeted clones were identified, eight of which were expanded and further analyzed by Southern blot as described above. All of these eight clones were confirmed as correctly targeted at both homology arms without additional random integrations (data not shown).

#### 2.1.3. Generation and molecular characterization of MDR1 humanized mice

For the generation of hMDR1 mice, four ES cell clones correctly targeted at the mouse *Mdr1a* and *Mdr1b* gene locus (described above), were expanded, injected into BALBc-blastocysts and transferred into foster mothers by TaconicArtemis as described previously [12]. Litters from these fosters were inspected visually and chimerism was determined by hair color. Highly chimeric animals were used for breeding to an efficient flipase (Flpe) deleter strain carrying a transgene that expresses Flpe in the germ line in order to delete the hygromycin and neomycin expression cassettes in the offspring (Fig 1E). The Flpe-deleter strain was generated by TaconicArtemis on a C57BL/6 genetic background. Offspring from chimeras derived from two of the *Mdr1a* and *1b* targeted ES cells were analyzed by PCR in order to determine the genotype of these mice. The following PCR primer pairs were used for the detection of the different alleles: (1) WT mouse *Mdr1a* allele: 5’-GTGATGGAACTTGAAGAGGACC-3’ and 5’-CACACACATTTGAAGGACTGC-3’, resulting in a 236 bp PCR fragment; (2) WT mouse *Mdr1b* allele: 5’-CCCCACAAACCAGACATAAGAGC-3’ and 5’-ATCTCTCTTAGCACCAGTCCAG-3’, resulting in a 396 bp PCR fragment; (3) Humanized *Mdr1a* allele: 5’-GGACTGTAACTGACTGCCTTGC-3’ and 5’-CGAAAGTGAAACAATCTGGCTAC-3’, resulting in a 490 bp PCR fragment and (4) Humanized *Mdr1b* allele: 5’-GGACTGTAACTGACTGCCTTGC-3’ and 5’-CCCTTTGCTTATCCTCGCC-3’, resulting in a 518 bp PCR fragment. The *Mdr1a* and *Mdr1b* targeted alleles segregated independently in offspring from chimeras derived from one of the two clones, suggesting that targeting had occurred on different alleles. In case of the other ES cell clone (A-F1) the targeted alleles co-segregated, indicating that the same alleles were targeted. Heterozygous mice derived from ES cell clone A-F1 were crossed by TaconicArtemis to generate homozygous hMDR1 mice. Homozygosity was determined by the presence of the corresponding humanized alleles and absence of the WT alleles using the PCR primers described above.

The hMDR1 mice were generated by a knock-in strategy as depicted in Fig 1, such that a cDNA of human *MDR1* followed by a polyA motif was introduced at the ATGs of both the mouse *Mdr1a* and *Mdr1b* genes by two consecutive rounds of targeting in mouse embryonic stem (ES) cells, resulting in double targeted ES cells (Fig 1A–1D). Transgenic mice from correctly targeted ES cells were generated subsequently. By further crosses with a mouse line expressing Flp-recombinase in the germ line, the hygromycin and neomycin expression cassette were deleted via recombination at the Flp recombinase recognition sites *frt* and *f3*, respectively (Fig 1E). Homozygous hMDR1 mice obtained by breeding appeared normal, could not be distinguished from WT animals, and had normal liver and body weights, survival rates and fertility (data not shown).

### 2.2. Animal husbandry

Mice were kept in accord with local laws and regulations and as described previously at breeding facilities [13]. Male FVB Mdr1a/1b(+/+) (FVB WT), FVB Mdr1a/1b(-/-) (Mdr1a/1b(-/-)), C57BL/6 Mdr1a/1b(+/+) (C57BL/6 WT) and C57BL/6 humanized MDR1 (hMDR1) mice of 10–11 weeks age were provided by Taconic Artemis (Cologne, Germany and Ejby, Denmark) to Uppsala University, Sweden. The mice were kept seven days in 12 hour light-dark cycle with free access to food and water before the study. The mice were housed two or three together, with free access to food and water. Occurrence of discord resulted in separation. During the experiment the mice were kept individually. These studies were approved by the Ethics Committee for Animal Research in Tierp, Sweden (Ref # C 21/098).

### 2.3. Chemicals

All chemicals were of analytical grade and all solvents were of high-performance liquid chromatography (HPLC) grade. Acetonitrile (Merck, Darmstadt, Germany), methanol (Merck, Darmstadt, Germany), formic acid (Merck, Darmstadt, Germany), acetic acid (Merck, Darmstadt, Germany), ammonia (Merck, Darmstadt, Germany), saline (Fresenius Kabi, Bad Homburg, Germany) and water (Milli-Q Academic system Millipore, Bedford, MA, USA) were used.

The following compounds were administered to the mice: Digoxin (Digoxin BioPhausia 0.25 mg/ml), docetaxel (Taxotere 20 mg/ml, Sanofi Aventis), verapamil (Verapamil Ratiopharm 2.5 mg/ml) and cyclosporine A (Sandimmun 50 mg/ml, Novartis) for parenteral use were obtained from Apoteket AB, Sweden. Quinidine gluconate was purchased from the Sigma-Aldrich Logistik GmbH, Schnelldrof, Germany. The compounds were diluted with saline to appropriate concentrations before administration. Elacridar (GF120918) (a gift from Pfizer, Sandwich, Kent, UK), was dissolved in dimethyl sulfoxide and then diluted with equal amount of saline before administration. Final infusion solution contained 50% dimethyl sulfoxide.

The following compounds were used in the chemical analysis of the samples: Digoxin (Fluka, Buchs, Switzerland purity > 98%), verapamil hydrochloride (Sigma Chemical Co., St. Louis, MO, USA, purity > 99%), methoxyverapamil hydrochloride (Sigma Chemical Co., St. Louis, MO, USA), docetaxel (Sigma Chemical Co., St. Louis, MO, USA purity > 97%), oxycodone (Apoteket AB Production and Laboratory, Sweden), D6-oxycodone (Cerilliant Corporation, Round Rock, TX, USA), elacridar (GF120918) (Pfizer, Sandwich, Kent, UK), and quinidine gluconate (Sigma-Aldrich Logistik GmbH, Schnelldorf, Germany > 98%).

Artificial extracellular fluid (ECF) was prepared on the day of the experiment. Composition of the working solution of ECF was glucose 1.802 g dissolved in 600 ml MQ water, stock ECF solution 100 ml, 280 mM CaCl_2_ 5 ml, 400 mM ascorbic acid 1ml, 10M NaOH 1.5 ml and MQ water to make volume up to 1000 ml. Finally pH was adjusted to 7.6. The stock ECF solution contained 129 mM NaCl, 3 mM KCl, 1.2 mM MgSO_4_*7H_2_O, 25 mM HEPES, 0.4 mM K_2_HPO_4_*2H_2_O in MQ water.

### 2.4. Study design for in vivo experiments

#### 2.4.1. BBB penetration studies

The P-gp substrates digoxin, verapamil and docetaxel, related to different chemical and therapeutic classes of drugs, were administered to study possible functional difference between human and mouse P-gp. Oxycodone was administered as an expected non-P-gp substrate (Boström et al., 2005). Brain to plasma total concentration ratios (K_p_) of the substances were measured for each animal. Unbound volume of distribution in brain (V_u, brain_) was measured with the brain slice method [14] and fraction unbound in plasma (f_u, p_) was measured with high throughput equilibrium dialysis.

All the substances were administered subcutaneously except docetaxel, which was given intravenously in the tail vein as a slow injection over five minutes due to its tissue irritating character. The doses administered were 1 mg/kg for digoxin, verapamil and oxycodone, respectively. The dose of docetaxel was 10 mg/kg. The volume administered of all substances was 0.01 ml/g_body weight. All substances other than oxycodone were given to five mice from each group i.e. Mdr1a/1b(-/-), FVB WT, hMDR1 and C57BL/6 WT mice. Oxycodone was given to three mice from each group.

Verapamil and docetaxel were also studied in combination with the P-gp inhibitor cyclosporine A in all four groups of mice. Cyclosporine A was administered intraperitoneally one hour before the drugs at a dose of 100 mg/kg.

The mice were sacrificed under anesthesia (2% isoflurane, nitrous oxide 1 L/min and medical air 1.5 L/min), one hour after administration of the drug. Blood was collected with a pre-heparinized syringe by cardiac puncture and was centrifuged for 5 minutes at 10000 rpm to obtain plasma. Brain was also sampled. The brain was divided in two equal parts and frozen on dry ice before weighed. All the samples were stored at -80°C until analysis.

#### 2.4.2. Plasma protein binding

Equilibrium dialysis was performed to measure the plasma unbound fraction of the drugs studied, and to measure possible differences in the plasma protein binding between hMDR1 and C57BL/6 WT mice. A high throughput, 96 well equilibrium dialysis apparatus (Model HTD96b, HTDialysis, Connecticut, USA) [15] with dialysis membrane strips with a molecular weight cut-off of 12–14 kDa was used. The concentrations of the substances were the same as those achieved in plasma during the in vivo experiments. One side of each well used was loaded with 125 μl of plasma spiked with drug and the other side was loaded with 125 μl phosphate buffer saline (PBS). The top surface of the Teflon block was then sealed with an adhesive film. Oxycodone and verapamil were dialyzed for 6 hours while digoxin and docetaxel was dialyzed for 24 hours, all at 37°C with a shaking speed of 115 rpm, on a Thermo Scientific MaxQ 4450 orbital shaker (Thermo Scientific, Waltham, Massachusetts USA). Triplicates of the same concentration for each compound were measured. After the incubation period 80 μl from the buffer side was added into 80 μl of blank mouse plasma and 80 μl from the plasma was added into 80 μl of PBS. Samples were stored at -20 until analysis.

#### 2.4.3. Nonspecific binding to brain parenchyma

To measure the volume of distribution for unbound drug in brain (V_u, brain_) in vitro, the high-throughput brain slice method was used [16, 17], with the main difference of mouse brain slices from four C57BL/6 WT mice rather than slices from rat brain was used. In short, six consecutive 300 μm brain slices on a coronal plane starting approximately 1.7 mm anterior from bregma (rostral striatum) up to approximately -0.1 mm anterior from bregma (caudal striatum) were cut. The cut slices were kept in plain ice-cold oxygenated ECF before the incubation. The six brain slices were transferred into one Ø80-mm flat-bottomed glass beaker containing 15 ml of buffer with the drugs digoxin, verapamil, docetaxel and oxycodone in cassette, each compound of 200 nM concentration in the buffer. Immediately after transferring the brain slices into the beaker, 200 μl of buffer was sampled as a reference for the brain slice viability test. The beaker was filled with 100% oxygen over the buffer, lightly covered with a glass lid and placed in a continuously oxygenated box inside a MaxQ4450 incubated shaker (Thermo Fisher Scientific, NinoLab, Sweden) for 5 h set at 37°C, with a rotation speed of 45 rpm. After 5 hours of incubation, the pH at 37°C was measured. A pH lower than 7.25 was one of the criteria of low viability of the brain slices. Before sampling the buffer, the beaker was placed resting for 5 minutes, which was required for sedimentation of the minor debris from the brain tissue. Two hundred μl of the buffer was thereafter aspirated and dispensed in an Eppendorf tube containing 200 μl of blank brain homogenate prepared with 4 volumes of buffer. The brain slices were removed, dried on filter paper, and weighed. Individual homogenization of the brain slices was made in 9 volumes (w/v) of ECF with an ultrasonic processor (VCX-130; Sonics, Chemical Instruments AB, Sweden). One brain slice was used for the detection of the maximum releasable lactate dehydrogenase (LDH) activity from the brain slice which indicates the viability of the cells in a brain slice [18, 19]. Samples were stored at -20°C pending LC-MS/MS analysis.

#### 2.4.4. P-glycoprotein inhibition studies using digoxin as a substrate

Inhibition of digoxin transport across the BBB by human and mouse P-gp was studied in C57BL/6 WT and hMDR1 mice. The mice were divided into 5 groups, each containing 5 humanized and 5 wild type mice. Digoxin was administered s.c. at a dose of 1 mg/kg as in the previous experiment. Blood and brain were sampled after 2 hours as described above. One group received digoxin only. The other 4 groups were given cyclosporine A 100 mg/kg i.p., quinidine 50 mg/kg i.p., verapamil 3 mg/kg s.c. or elacridar 3 mg/kg s.c., one hour before the digoxin administration. The volumes administered were 0.01 ml/g body weight, apart from elacridar, which was administered in a volume of 0.005 ml/g.

#### 2.4.5. Effect of P-glycoprotein on the BBB transport of oxycodone

The influence of P-gp on the transport of oxycodone across the BBB was studied in male C57BL/6 WT mice (n = 20) with and without the P-gp blockers cyclosporine A 100 mg/kg i.p, verapamil 3 mg/kg s.c. or elacridar 6 mg/kg s.c. They were divided into 4 groups of 5 mice each. One group was administered oxycodone only. For the other three groups the P-gp blockers were administered one hour before the administration of oxycodone, and sampling of blood was done under anesthesia one hour after the oxycodone administration as described above. Brain and liver were sampled after sacrificing the animal.

### 2.5. Chemical analysis

#### 2.5.1. Plasma sample treatment

The plasma samples were thawed in room tempered water. They were mixed by vortex (Uniequip Laborgeräte, Martinsried) and centrifuged for one minute at 10 000 rpm (Force 7, Denver Instrument Company, Denver, USA). Fifty μl of the samples were transferred to polypropylene Eppendorf tubes. The proteins were precipitated by adding 150 μl acetonitrile. For the verapamil analysis, the acetonitrile contained methoxyverapamil as internal standard at a concentration of 200 ng/ml. For the oxycodone analysis, the acetonitrile contained D6-oxycodone as internal standard at a concentration of 30 ng/ml. The samples were vortexed and centrifuged for three minutes at 10000 rpm. The analysis of digoxin, docetaxel, quinidine and elacridar did not contain an internal standard.

For digoxin and docetaxel 100 μl of the supernatant was added to 200 μl ammonium formate buffer and 100 μl ammonium acetate buffer in Eppendorf tubes, respectively. For verapamil and elacridar 50 μl of the supernatant was added to 1000 μl mobile phase, and for quinidine in 500 μl of mobile phase. The samples were vortexed and centrifuged during one minute at 10000 rpm. Fifty μl of the samples were then transferred to vials and covered with parafilm. Twenty μl was injected onto the LC-MS/MS system. For oxycodone and quinidine 40 μl and 25 μl of supernatant was transferred to a vial, covered with parafilm, 20 μl and 10μl respectively were injected directly onto the LC-MS/MS system.

Cyclosporine A was analyzed in whole blood. The samples were vortexed and 50 μl was transferred to Eppendorf tubes. The proteins were precipitated by adding 100 μl acetonitrile. The samples were vortexed and left for five minutes before they were vortexed again and centrifuged during three minutes. Fifty μl of the supernatant was added to 500 μl mobile phase, vortexed and centrifuged. Fifty μl was transferred to a vial, covered with parafilm and 20 μl was injected onto the LC-MS/MS system.

For the analysis of equilibrium dialysis samples similar methods as for the plasma sample treatment were used with some minor volume adjustments.

#### 2.5.2. Brain sample treatment

The brains were thawed on an ice bath at 0–2°C. A 4-fold volume (w/v) of ice-cold saline was added before homogenization (Heidolph R2R 2020, Schwabach, Germany). The homogenate was again frozen until sample preparation and analysis. The homogenate samples were thawed in a 4°C water bath. They were vortexed and 200 μl of the samples were transferred to Eppendorf tubes that were put in an ice bath. The proteins were precipitated by adding 600 μl ice-cold acetonitrile.

For verapamil and oxycodone the acetonitrile contained internal standard at a concentration of 5 and 6 ng/ml of methoxyverapamil and oxycodone-D6, respectively. The samples were vortexed and put in an ultrasonic bath (Branson 3210, Branson Ultrasonic Corporation, Danbury, USA) for ten minutes. The samples were again vortexed and centrifuged for five minutes at 10000 rpm.

For digoxin and docetaxel the acetonitrile did not contain an internal standard. After precipitation of proteins, 200 μl of the supernatant was transferred to an Eppendorf tube. It was evaporated to dryness under a flow of nitrogen at 40°C and re-dissolved in 200 μl mobile phase. For verapamil 200 μl of the supernatant was added to 200 μl 0.2% formic acid in an Eppendorf tube. The samples were vortexed and centrifuged for one minute before 50 μl was transferred to new vials, covered with parafilm and 20 μl was injected onto the LC-MS/MS system, except for docetaxel where 40 μl was injected. For oxycodone 40 μl of the supernatant was transferred to a vial, covered with parafilm and 20 μl was injected directly onto the LC-MS/MS system.

#### 2.5.3. Drug quantification

The samples were analyzed with reversed phase liquid chromatography (LC) in combination with tandem mass spectrometry, LC-MS/MS, equipped with a Shimadzu LC-10ADvp pump (Shimadzu, Kyoto, Japan), an SIL-HTc pump (Shimadzu, Kyoto, Japan) and a Quattro Ultima detector (Waters, Milford, MA, USA). The column used for digoxin and verapamil was a HyPurity C18, 3μm particle size, 50 x 4.6 mm + precolumn 10x4 mm. For docetaxel, oxycodone and cyclosporine A the column was a Zorbax Eclipse XDB-CN, 5μm particle size, 150 x 4.6 mm. Analytical column used for quinidine and elacridar was Gemini C18, 3μm particle size, 100 x 4.6 mm (Phenomenex). The mobile phase used for digoxin was acetonitrile (ACN): 5mM ammonium formate buffer pH 3.4 (26: 74), for verapamil it was 40% ACN in 0.2% formic acid, for docetaxel 50% ACN in 2mM ammonium acetate pH 5.0, for oxycodone 45% ACN on 5 mM ammonium acetate and for cyclosporine A ACN: 5 mM ammonium formate buffer pH 3.4 (50:50). Mobile phase used for quinidine was 10 mM ammonium acetate pH 10: ACN (45:55) and for elacridar it was 10 mM ammonium acetate pH 10: ACN (30:70). The MassLynx software (Micromass UK Limited, Manchester, UK) was used to monitor the analysis. The analysis was performed in positive electrospray ionization (ESI+) mode. The conditions for the LC varied for the different substances. For digoxin, verapamil and methoxyverapamil, the flow rate was 0.8 ml/min while for all other drugs it was 1 ml/min. Retention time for digoxin, verapamil, methoxyverapamil, docetaxel, oxycodone, D6 oxycodone, quinidine, elacridar and cyclosporine A were 4.4, 2.4, 2.7, 4.2, 6.4, 6.5, 2.3, 3.2 and 5.2 min, respectively. Tuning was performed to find optimal settings for the MS/MS detector. Source temperature was set to 130 C for verapamil, methoxyverapamil, quinidine, elacridar and cyclosporine A and 120 C for all other compounds. Desolvation temperature was set to 350 C for digoxin, 300 for docetaxel and 450 for the other compounds. For digoxin, verapamil, methoxyverapamil, docetaxel, elacridar, oxycodone, quinidine and cyclosporine A capillary voltage was 3.5, 1.4, 1.4, 3.8, 1.5, 0.9, 0.9 and 3.2 kV while cone voltage was 40, 70, 70, 25, 110, 55, 40 and 140 V, respectively. Desolvation gas (N_2_) and cone gas (N_2_) were set to 1000 and 200 L/h for all compounds except digoxin and docetaxel for which cone gas (N_2_) was set to 250 L/h. Finally the precursor-product ion pair for digoxin, verapamil, methoxyverapamil, docetaxel, oxycodone, quinidine, elacridar and cyclosporine A were *m/z* 798.35–651.4, 455.2–165.0, 485.3–165.0, 808.25–527.3, 315.8–297.7, 321.8–303.7, 325–306.9, 564.3–251.8 and 1202.8–1185 respectively.

### 2.6. Calculations

The total brain concentration (ng/g brain tissue) of the substance was divided with the total plasma concentration (ng/ml) of the substance to obtain the tissue partition ratio (K_p_), which was compared between the groups of mice. The brain concentration was corrected for residual blood volume using Eqs 1 and 2 using the values of apparent vascular space of plasma proteins (V_protein)_ and apparent vascular space of plasma water (V_water_) reported by Friden et al [20]

$$A_{brain}=\frac{C_{brain,h}-V_{eff}\times C_{plasma}}{1-V_{water}}$$ (1)

$$V_{eff}=f_{u,p}\times V_{Water}+(1-f_{u,p})\times V_{protein}$$ (2)

A_brain_ is the amount of the drug in brain tissue, V_eff_ is effective plasma space in the brain for a given drug, C_brain, h_ is the brain homogenate concentration and C_plasma_ is the total plasma concentration of the drug. The the fraction unbound in plasma (f_u, p)_ measured with equilibrium dialysis was used in the calculations.

By using K_p_, the unbound volume of distribution in brain, V_u, brain_, describing the total amount in brain tissue vs the unbound brain interstitial fluid concentratioin, and the fraction unbound in plasma, f_u, p_ values, the K_p, uu_ (ratio of unbound drug in brain to unbound drug in plasma) was calculated [21] by

$$K_{p,uu}=\frac{K_{p}}{V_{u,brain}\times f_{u,p}}$$ (3)

Unpaired, two-tailed Student’s *t*-test was performed to capture any differences between brain distributions of drugs between different groups. The significance level was set to 5% (α = 0.05).

### 2.7. In vitro analysis of expression levels of transporters in brain capillaries

#### 2.7.1. Preparation of brain capillary rich fraction pooled from 10 mice

10 frozen cerebrums from wild-type C57BL/6 or hMDR1 mice were dissected into 1-mm pieces, and homogenized with a Potter—Elvehjem homogenizer using 20 up and down no-rotated strokes by hand in 5 volumes of solution B (101 mM NaCl, 4.6 mM KCl, 2.5 mM CaCl_2_, 1.2 mM KH_2_PO_4_, 1.2 mM MgSO_4_, 15 mM HEPES, pH 7.4) per brain weight. The homogenate was centrifuged at 1,000g for 10 min, the pellet was suspended in solution B containing 16% dextran, and the suspension was centrifuged for 15 min at 4500 g. The resulting pellet was suspended in solution A (solution B containing 25 mM NaHCO_3_, 10 mM glucose, 1 mM pyruvate and 5g/L bovine serum albumin), passed through a 210 μm nylon mesh, and then washed with 10 mL of solution A. The solution passing through the 210 μm mesh including wash solution was passed through a 85 μm nylon mesh, and then washed with 10 mL of solution A. The solution passing through the 85 μm mesh including wash solution was passed through a 20 μm nylon mesh, and then washed with 40 mL of solution A. The capillaries retained on the 20 μm nylon mesh were immediately collected using solution A, centrifuged at 1000g for 5 min, and then suspended in solution B. The capillaries were again centrifuged at 1000g for 5 min, and then suspended in solution B. The capillaries were again centrifuged at 1000g for 5 min, suspended in hypotonic buffer (10 mM Tris-HCl, 10 mM NaCl, 1.5 mM MgCl_2_, pH7.4), sonicated, and then stored at -80°C after measurement of the protein concentration by the Lowry method using the DC protein assay reagent (Bio-Rad, Hercules, CA). All procedures were carried out at 4°C.

#### 2.7.2. Multiplexed SRM/MRM analysis in LC-MS/MS

Protein expression amounts of the target molecules were simultaneously determined by means of multiplexed selected/multiple reaction monitoring (SRM/MRM) analysis as described previously [1]. Whole tissue lysates of isolated brain capillaries (50 μg protein) were solubilized in 500 mM Tris—HCl (pH 8.5), 7 M guanidine hydrochloride, 10 mM EDTA, and the proteins were S-carbamoylmethylated as described [1]. The alkylated proteins were precipitated with a mixture of methanol, chloroform and water. The precipitates were dissolved in 6 M urea in 100 mM Tris—HCl (pH 8.5), diluted 5-fold with 100 mM Tris—HCl (pH 8.5) and treated with lysyl endopeptidase (LysC; Wako Pure Chemicals, Osaka, Japan) at an enzyme/substrate ratio of 1:100 and 0.05% ProteaseMax (Promega, Madison, WI) at 25°C for 3 h, followed by treatment of TPCK-treated trypsin (Promega) at an enzyme/substrate ratio of 1:100 at 37°C for 16 h. The tryptic digests were mixed with internal standard peptides and formic acid, and then centrifuged at 4°C and 17,360 g for 5 min. The supernatants were subjected to HPLC or microLC-MS/MS analysis.

The HPLC-MS/MS analysis was performed by coupling an Agilent 1200 HPLC system (Agilent Technologies, Santa Clara, CA) to a triple quadrupole mass spectrometer (QTRAP5500; AB SCIEX, Foster City, CA) equipped with Turbo V ion source (AB SCIEX) and 65 μm ID electrode (AB SCIEX). Samples equivalent to 33.3 μg protein were injected onto a Waters XBridge BEH130 C18 (1.0 × 100 mm, 3.5 μm) column together with 500 fmol of internal standard peptides. Mobile phases A and B consisted of 0.1% formic acid in water and 0.1% formic acid in acetonitrile, respectively. The peptides were separated and eluted from the column at room temperature using a linear gradient with a 130-min run time at a flow rate of 50 μL/min. The sequence was as follows: (A:B), 99:1 for 5 min after injection, 40:60 at 65 min, 0:100 at 66 min and up to 68 min, 99:1 at 70 min and up to 130 min.

The microLC-MS/MS analysis was performed by coupling an ekspert microLC 200 system (Eksigent, Dublin, CA) to the QTRAP5500 equipped with Turbo V ion source and 25 μm ID electrode (AB SCIEX). Samples equivalent to 6.73 μg protein were injected onto a HALO Fused-Core C18 (0.3 × 100 mm, 2.7 μm) column (Eksigent) together with 50 fmol of internal standard peptides. Mobile phases A and B consisted of 0.1% formic acid in water and 0.1% formic acid in acetonitrile, respectively. The peptides were separated and eluted from the column at 40°C using a linear gradient with a 55-min run time at a flow rate of 10 μL/min. The sequence was as follows: (A:B), 99:1 for 2 min after injection, 50:50 at 32 min, 0:100 at 35 min and up to 37 min, 99:1 at 39 min and up to 55 min.

The eluted peptides were simultaneously and selectively detected by means of electro-spray ionization in a multiplexed SRM/MRM analysis. The dwell time was 8 to 25 msec per SRM/MRM transition. Each molecule was monitored with four sets of SRM/MRM transitions (Q1/Q3-1, Q1/Q3-2, Q1/Q3-3, Q1/Q3-4) derived from one set of standard and internal standard peptides. Mouse Mdr1a-specific and mouse Mdr1b-specific peptides were monitored with the SRM/MRM transitions listed in S1 Table. The other molecules were monitored with the peptides and SRM/MRM transitions reported by Kamiie *et al*.[22], Uchida *et al*. [1] and Agarwal *et al*. [23]. Signal peaks detected at the same retention time as an internal standard peptide were defined as positive. When positive peaks were observed in three or four sets of SRM/MRM transitions, the molecules were considered to be expressed in brain capillaries, the protein expression levels were determined as the average of the quantitative values obtained from the different SRM/MRM transitions in three measurements, and then shown as the mean ± S.E.M. The S.E.M. represents the variability of protein expression level determined in different MS/MS transitions in three analyses, but does not represent the inter-individual variability. The limit of quantification was calculated as described previously [1].

## Results

### 3.1. Assessment of P-gp transporter function

**Fig 2:**
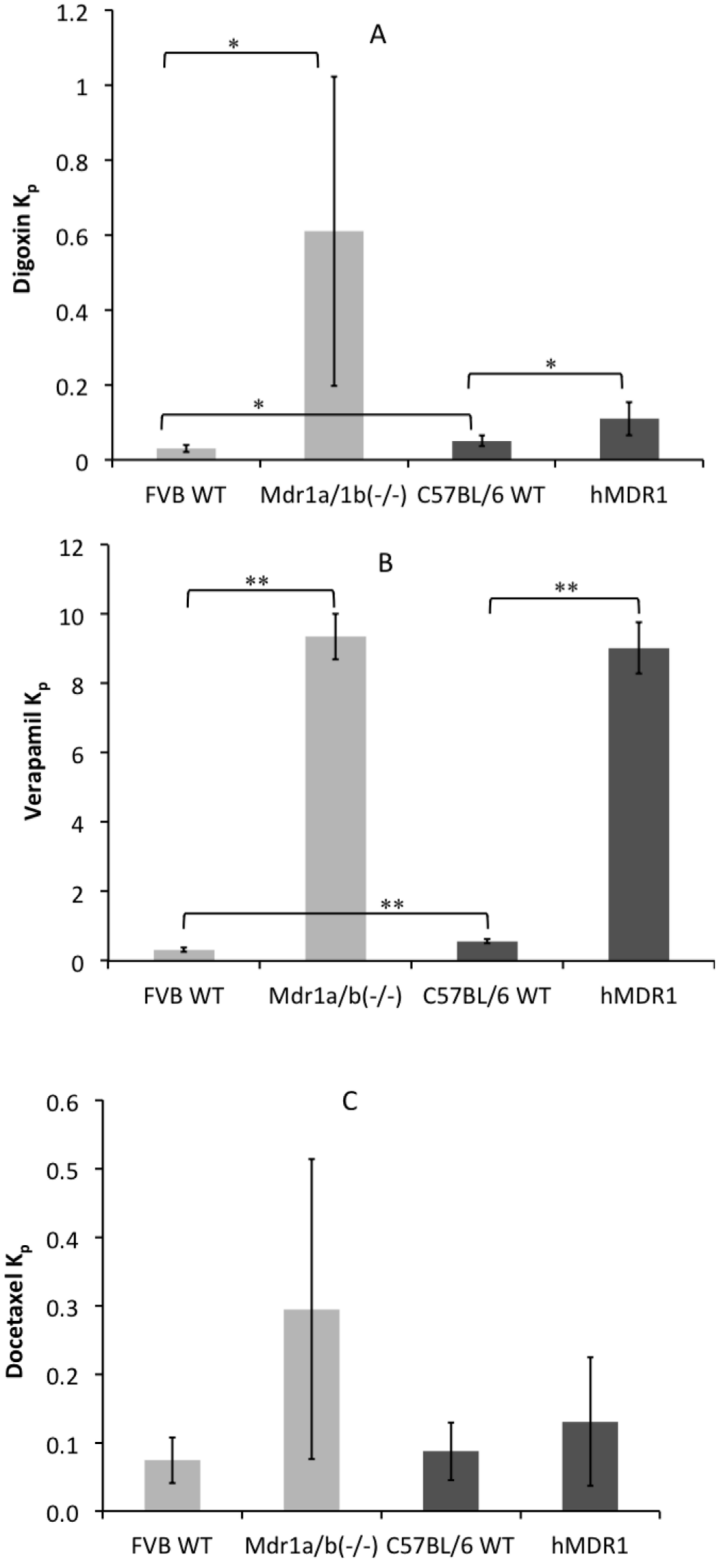
Comparison of K_p_ between the Mdr1a/1b(-/-) mice with FVB WT controls and hMDR1 mice with the C57BL/6 WT controls for digoxin (A) verapamil (B) and docetaxel (C) (* p < 0.05, ** p < 0.01). The doses of 1 mg/kg for digoxin and verapamil were administered subcutaneously while 10mg/kg of docetaxel was administered in tail-vein as a slow injection over five minutes due to its irritating character (n = 5 for each group).

The K_p_ values for digoxin, verapamil, and docetaxel were 20, 30 and 4 times higher, respectively, in the Mdr1a/1b(-/-) mice than in the FVB WT controls, confirming the role of P-gp for the efflux of these drugs at the BBB (Fig 2A, 2B and 2C). The K_p_ values for digoxin, verapamil and docetaxel were 2, 16 and 2 times higher in the hMDR1 compared to C57BL/6 WT mice, respectively (Fig 2A, 2B and 2C). This indicates either a difference in P-gp substrate specificity or expression level at the BBB between the hMDR1 and C57BL/6 WT mice. Apart from these differences, the FVB WT mice had significantly lower K_p_ compared to the C57BL/6 WT mice for both digoxin and verapamil (Fig 2A and 2B) showing that also the two wild type strains of mice have differences in the BBB transport of these two drugs.

**Fig 3:**
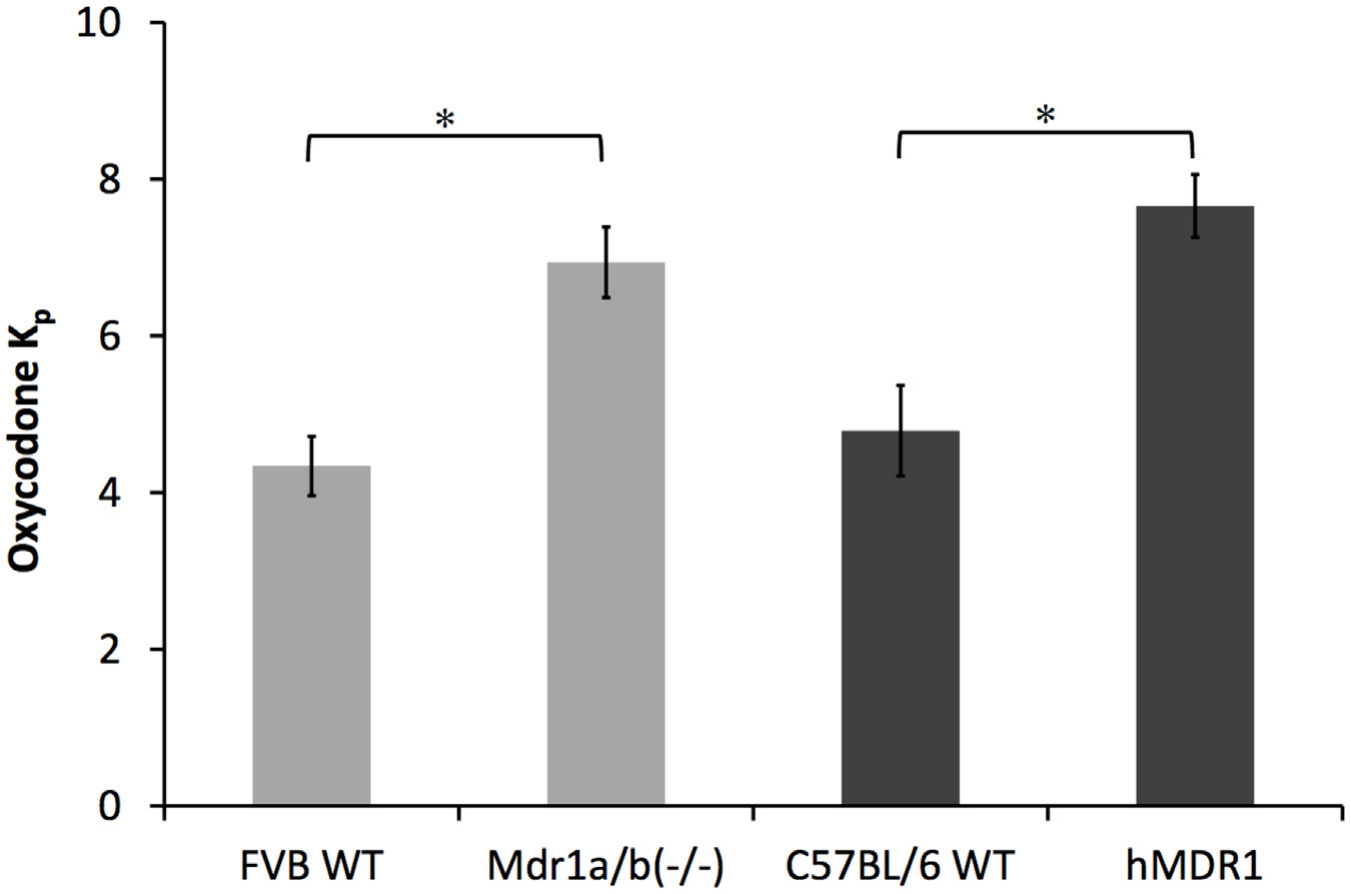
Comparison of K_p_ between the Mdr1a/1b(-/-) mice with FVB WT controls and hMDR1 mice with C57BL/6 WT controls for oxycodone (* p < 0.05, ** p < 0.01). Oxycodone was administered 1 mg/kg subcutaneously (n = 3).

For oxycodone, which was considered not to be a substrate of P-gp, both the hMDR1 and Mdr1a/1b(-/-) mice had 1.6 times higher K_p_ than the corresponding wild type mice (Fig 3). Results from further experiments combining oxycodone and blockers are presented below.

The nonspecific binding to brain parenchymal tissue, described by V_u, brain_, was very different for the four drugs, and was 45, 48, 789 and 4.0 ml*g_brain^-1^ for digoxin, verapamil, docetaxel and oxycodone, respectively, estimated with the brain slice method (Table 1). The K_p, uu_ was calculated based on K_p_ and V_u, brain_ values together with f_u, p_ (Table 1). For all four compounds there was a trend towards a higher K_p, uu_ in the hMDR1 mice compared to the C57BL/6 WT controls (Table 1), showing differences in the function or expression level of human and mouse P-gp in the two models. For oxycodone the K_p, uu_ was calculated to be 1.9 in C57BL/6 WT mice and 3.0 in the hMDR1 mice confirming the presence of active uptake transport at the BBB also in mice. A very interesting finding was that the K_p, uu_ for verapamil was also higher than unity (i.e. 1.6) in hMDR1 mice, reflecting the likely presence of active uptake transport also for verapamil at the BBB. This was observed in both the hMDR1 and Mdr1a/1b(-/-) mice.

**Table 1:** K_p, uu_ values of the C57BL/6 WT mice (WT) and hMDR1 mice calculated using V_u, brain_ and f_u, plasma_ measured with the brain slice method and equilibrium dialysis respectively.

Drug	Mice type	K_p_	V_u, brain_ (ml/g_brain)	f_u(plasma)_	K_p, uu_
Digoxin	WT	0.05	44.8	0.594	0.002
	hMDR1	0.11		0.607	0.004
Verapamil	WT	0.6	48.2	0.115	0.108
	hMDR1	9		0.117	1.60
Docetaxel	WT	0.09	789	0.053	0.002
	hMDR1	0.13		0.052	0.003
Oxycodone	WT	4.8	3.75	0.681	1.88
	hMDR1	7.7		0.681	3.02

**Fig 4:**
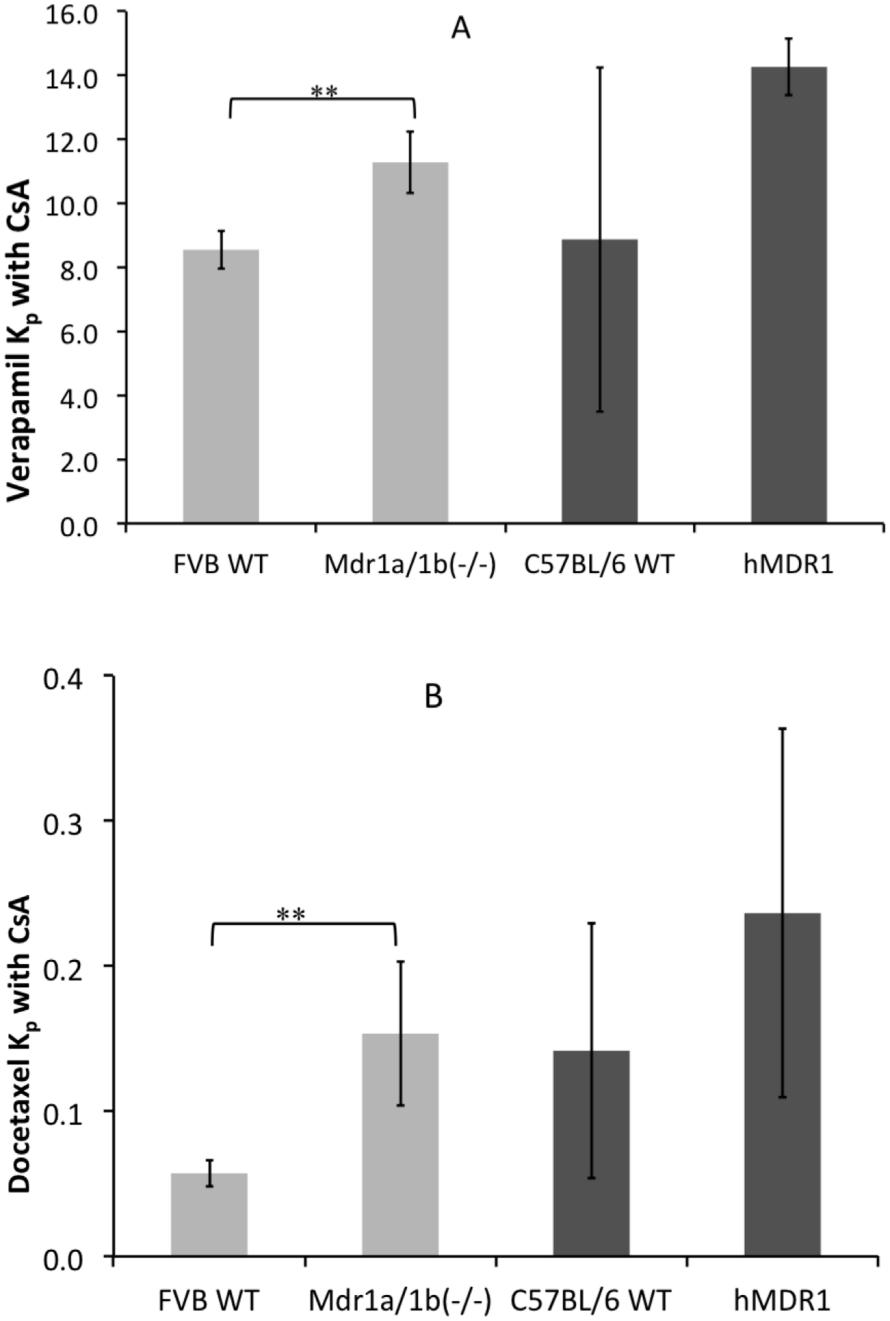
Comparison of K_p_ between the Mdr1a/1b(-/-) mice with FVB WT controls and hMDR1 mice with C57BL/6 WT controls with their wild type for verapamil (A) and docetaxel (B) in the presence of cyclosporine A (* p < 0.05, ** p < 0.01). Cyclosporine A was administered intraperitoneally one hour before the drugs at a dose of 100 mg/kg (n = 5 for each group).

For verapamil and docetaxel, differences in the P-gp transport function were also studied in the presence of cyclosporine A, being a broader blocker of several transporters including P-gp. In Mdr1a/1b(-/-) mice the K_p_ of verapamil and docetaxel were 1.3 and 2.7 times higher, respectively, than in the FVB WT mice in the presence of cyclosporine A (Fig 4A and 4B). In the hMDR1 mice, the K_p_ was 1.3 and 1.7 times higher compared to in the C57BL/6 WT mice for the two drugs (Fig 4A and 4B). The cyclosporine A blood concentrations achieved in the different mouse strains were the same and are presented in Table 2.

**Table 2:** Cyclosporine A concentrations (μg/ml) in blood of the different groups of mice. The first column represents the drug studied in the presence of cyclosporine A.

Drug studied	FVB WT mice	Mdr1a/1b(-/-) mice	C57BL/6 WT mice	hMDR1 mice
Verapamil	28.8 ± 4.8	35.2 ± 10.8	37.9 ± 17	35.4 ±6.9
Docetaxel	29.2 ± 9.9	29.6 ± 16.9	16.5 ± 9.2	22.3 ±5.7

### 3.2. P-glycoprotein inhibition studies

**Fig 5:**
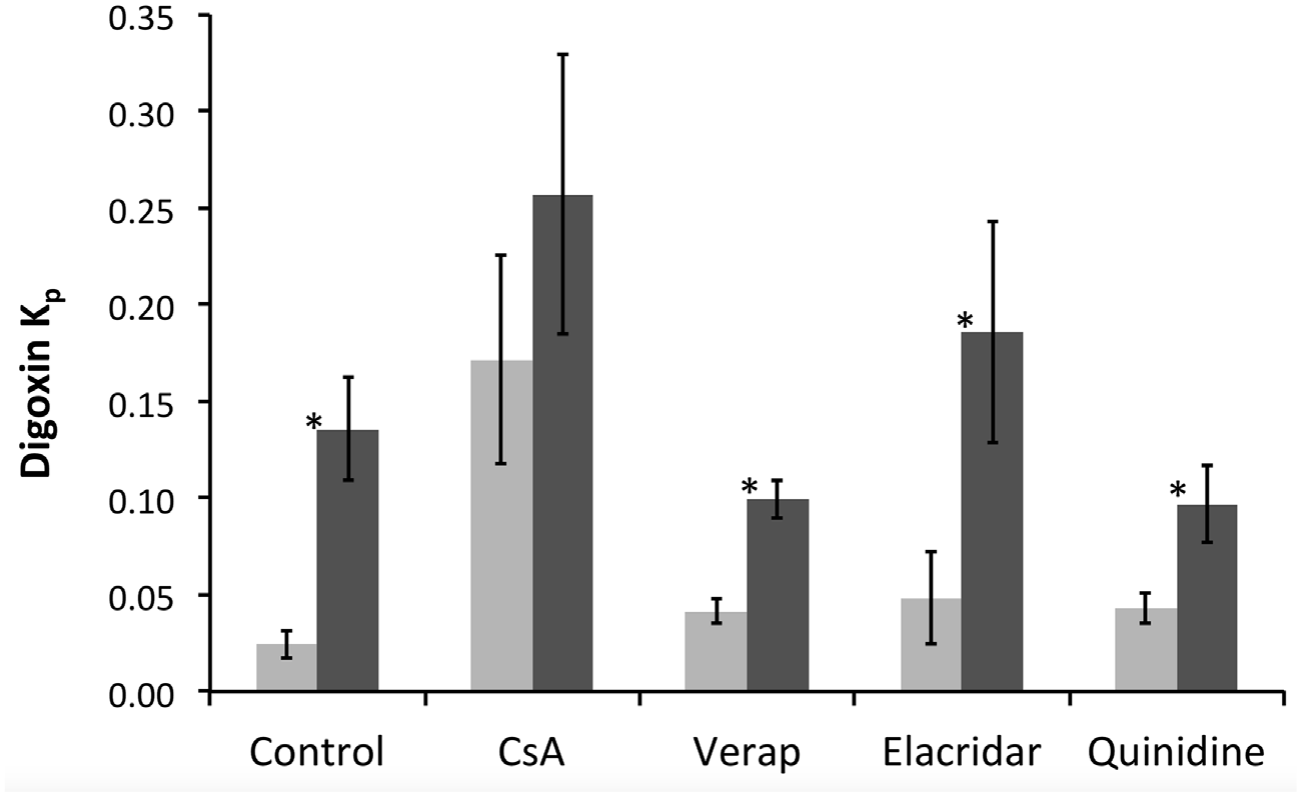
K_p_ values of digoxin in C57BL/6 WT (n = 5 for each group) (grey bars) and hMDR1 mice (n = 5) (black bars) in the presence of different blockers (* p < 0.05). Digoxin was administered subcutaneously at a dose of 1 mg/kg. P-gp blockers were given as cyclosporine A 100 mg/kg intraperitoneally, quinidine 50 mg/kg intraperitoneally, verapamil 3 mg/kg subcutaneously or elacridar 3 mg/kg subcutaneously, one hour before the digoxin administration.

Digoxin had a significantly higher K_p_ in hMDR1 mice as compared to the C57BL/6 WT controls, also when the mice were treated with different blockers (verapamil, elacridar and quinidine) with the exception of cyclosporine A (Fig 5). The ratio of digoxin K_p_ with and without blockers was 1.7 to 7.0 in C57BL/6 WT mice while in hMDR1 mice it was 0.7 to 1.7 in the presence of the different blockers (Table 3). This further reveals differences in the way the different blockers affected the brain distribution of digoxin between humanized and wild type mice (Table 3). The plasma concentrations of the blockers were also analyzed and are presented in Table 4. Chemical analysis showed that the concentrations of P-gp blockers were not statistically different in the different groups.

**Table 3:** The ratio* of K_p_ with blocker/K_p_ in control mice for digoxin, representing a difference in the effect of the blockers between C57BL/6 WT and hMDR1 mice.

Mouse Group	Blocker used			
	Cyclosporine A	Verapamil	Elacridar	Quinidine
C57BL/6 WT	7.00	1.69	1.97	1.76
hMDR1	1.89	0.73	1.37	0.71

(* No variability on ratio as mean K_p_ values were used to calculate it).

**Table 4:** Blood levels of the blockers used when studying digoxin transport in C57BL/6 WT and hMDR1 mice.

P-gp Blocker	C57BL/6 WT mice	hMDR1 mice
Cyclosporine A (μg/ml)	38.6 ± 10.4	40.4 ± 10.5
Verapamil (ng/ml)	80 ± 12	65.2 ± 18.2
Elacridar (ng/ml)	140 ± 97	190 ± 34
Quinidine (ng/ml)	520 ± 290	470 ± 68

### 3.3. Effect of P-glycoprotein on BBB transport of oxycodone

**Fig 6:**
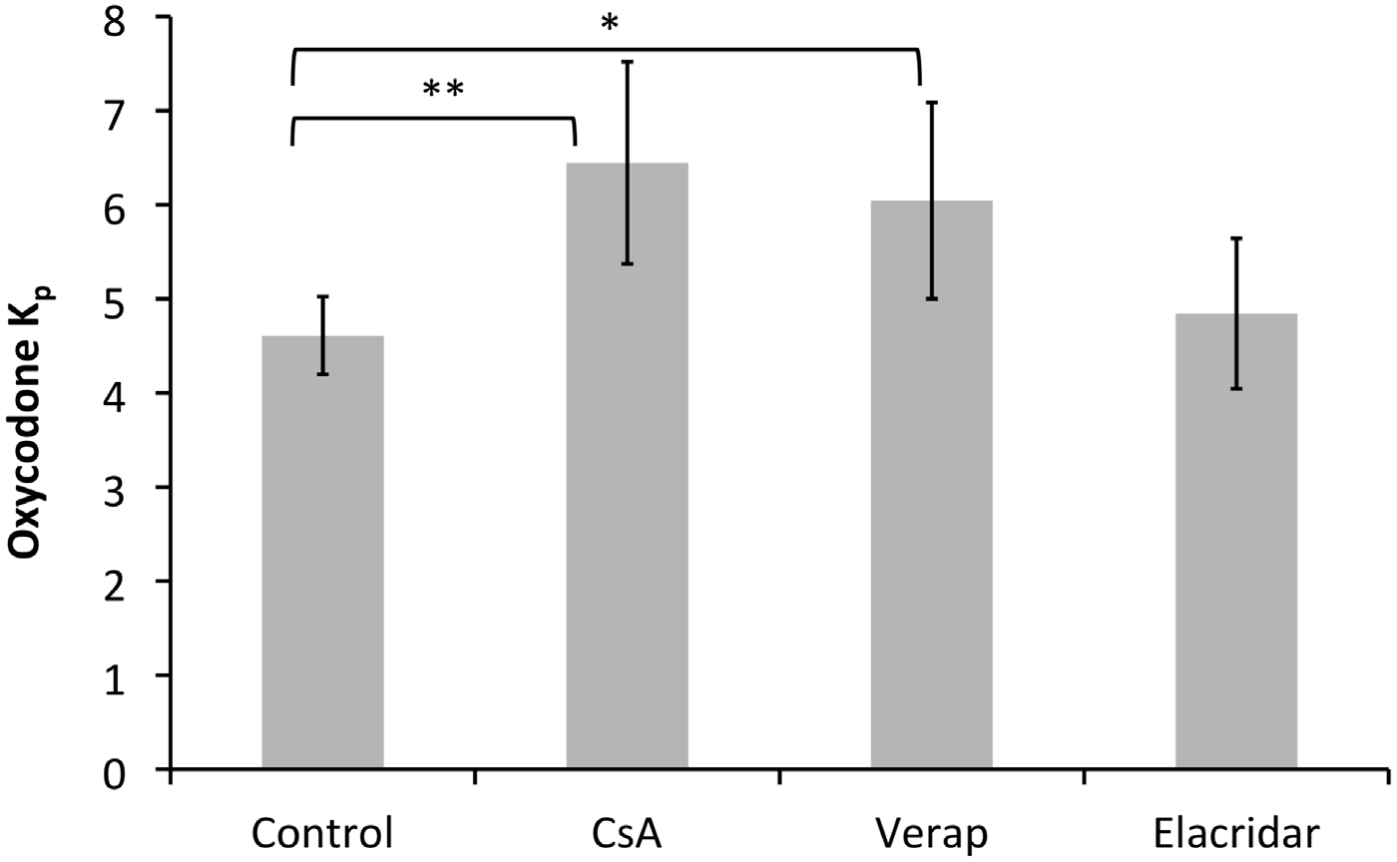
Effect of different P-gp blockers on oxycodone K_p_ in C57BL/6 WT mice (n = 20), (* p < 0.05, ** p < 0.01). Oxycodone was administered 1 mg/kg subcutaneously. The P-gp blockers were given cyclosporine A 100 mg/kg intraperitoneally, verapamil 3 mg/kg or elacridar 6 mg/kg subcutaneously one hour before the administration of oxycodone.

The K_p_ of oxycodone unexpectedly and significantly increased when C57BL/6 WT mice were treated with cyclosporine-A and verapamil (Fig 6). However, the relatively specific P-gp blocker elacridar (GF120918) had no effect on the brain distribution of oxycodone showing minimal involvement of P-gp and/or BCRP in the efflux of oxycodone from the brain (Fig 6).

### 3.4. Quantitative targeted absolute proteomics (QTAP) for membrane proteins in the isolated brain capillaries

As shown in Table 5, the hMDR1 protein was observed in the isolated brain capillaries from hMDR1 mice, with protein levels 62.8-fold smaller than those of Mdr1a in C57BL/6 WT mice. When compared to the reported data of QTAP analysis in human brain microvessels [1], the protein expression levels of hMDR1 in hMDR1 mice were 17.1-fold smaller than those in the human BBB. Furthermore, mouse Mdr1a proteins were also detected in the hMDR1 mice, with protein expression levels 6.90-fold greater than those of hMDR1, although the levels were 9.10-fold smaller than those of Mdr1a in C57BL/6 WT mice.

**Table 5:** Protein expression levels of membrane proteins in brain capillaries isolated from C57BL/6 WT and hMDR1 mice.

Molecular Names	Protein expression level (fmol/μg protein)	
	C57BL/6 WT mice	hMDR1 mice
**P-gp**		
Human ABCB1 (Human MDR1) ^a^	U.L.Q.(< 0.112)	0.355 ± 0.068
Mouse abcb1a (mouse Mdr1a) ^b^	22.3 ± 2.9	2.45 ± 0.19 *
ABCB1 & abcb1a (MDR1 & Mdr1a) ^c^	25.3 ± 1.5	2.60 ± 0.33 *
Mouse abcb1b (mouse Mdr1b)	U.L.Q.(< 0.136)	U.L.Q.(< 0.134)
**ABC transporters**		
Abca1	U.L.Q.(< 0.156)	U.L.Q.(< 0.261)
Abca2	U.L.Q.(< 0.624)	U.L.Q.(< 0.779)
Abca8a	U.L.Q.(< 0.213)	U.L.Q.(< 0.461)
Abca8b	U.L.Q.(< 0.184)	U.L.Q.(< 0.166)
Abcc1 (Mrp1)	U.L.Q.(< 0.302)	U.L.Q.(< 0.223)
Abcc3 (Mrp3)	U.L.Q.(< 0.0650)	U.L.Q.(< 0.0620)
Abcc4 (Mrp4)	1.33 ± 0.19	1.81 ± 0.21
Abcc5 (Mrp5)	U.L.Q.(< 0.400)	U.L.Q.(< 0.363)
Abcc6 (Mrp6)	U.L.Q.(< 0.124)	U.L.Q.(< 0.151)
Abcc10 (Mrp7)	U.L.Q.(< 1.04)	U.L.Q.(< 1.06)
Abcg2 (Bcrp)	8.69 ± 0.82	5.67 ± 0.14
**SLC transporters and accessary proteins**		
Slc2a1 (Glut1)	194 ± 13	134 ± 2
Slc7a5 (Lat1)	1.08 ± 0.29	0.854 ± 0.170
Slc3a2 (4F2hc)	23.5 ± 3.7	20.1 ± 0.5
Slc16a1 (Mct1)	18.8 ± 0.5	9.72 ± 0.53 *
Cd147	19.6 ± 1.3	13.1 ± 1.5
Slc21a14 (Oatp14)	2.90 ± 0.36	2.45 ± 0.46
Slc22a8 (Oat3)	2.74 ± 0.21	3.05 ± 0.30
Slc29a1 (Ent1)	2.82 ± 0.22	2.88 ± 0.21
Slc29a4 (Pmat)	U.L.Q.(< 0.0990)	U.L.Q.(< 0.0990)
**Other transporter**		
Rlip76	U.L.Q.(< 0.101)	U.L.Q.(< 0.0990)
**Receptors**		
Insr	1.09 ± 0.06	0.972 ± 0.121
Lrp1	1.82 ± 0.19	2.13 ± 0.26
Tfr1	4.91 ± 0.30	3.13 ± 0.23 *
**Tight junction protein**		
Claudin-5	10.0 ± 1.7	8.09 ± 0.43
**Marker proteins**		
Na^+^/K^+^ ATPase	60.7 ± 2.4	99.0 ± 6.3 *
Gamma-gtp	3.96 ± 0.13	2.54 ± 0.18 *

Brain capillary was isolated from the pooled frozen brains of 10 mice by means of nylon mesh method. Brain capillary passing through an 85 μm nylon mesh and retained on a 20 μm nylon mesh was collected, and digested with lysyl endopeptidase and trypsin. The digest was subjected to LC-MS/MS analysis with internal standard peptides. The each target peptide was monitored with four different SRM/MRM transitions. The preparation of pooled brain capillary from 10 mice was conducted only one time. For the pooled brain capillary, the digestion followed by LC-MS/MS analysis was repeated three times. The protein expression level of target molecule was determined as an average and variability (mean ± S.E.M.) of the quantitative values obtained from different SRM/MRM transitions in three analyses. Therefore, the S.E.M. represents the variability of protein expression level determined in different MS/MS transitions in three analyses, but does not represent the inter-individual variability.

U.L.Q., under the limit of quantification.

*p < 0.01, significantly different from the C57BL/6 WT mice.

^a^ measured by using a peptide probe set specific for human MDR1.

^b^ measured by using a peptide probe set specific for mouse mdr1a.

^c^ measured by using a peptide probe set common for human MDR1 and mouse mdr1a.

For the other membrane proteins, the protein expression levels were not different between the hMDR1 and C57BL/6 WT mice, although mct1, tfr1, Na^+^/K^+^ ATPase and gamma-gtp showed statistically significant differences. This suggested that there was no significant difference in the purity of the isolated brain capillaries and the efficiency of LysC/trypsin digestion between the hMDR1 and C57BL/6 WT mice.

## Discussion

The present study evaluated the hMDR1 mouse model by integrating the selective absolute quantification of human MDR1, mouse Mdr1a and Mdr1b protein expressions combined with the in vivo functional analysis to evaluate the P-gp efflux activities for P-gp substrates at the BBB. The hMDR1 mice were found to have higher brain delivery of all drugs studied (digoxin, verapamil, docetaxel and oxycodone), indicating that these drugs were effluxed to a lower extent at the BBB of the hMDR1 mice than in their C57BL/6 WT controls. The species-specific absolute quantification of protein expression by the QTAP analysis clarified that both human MDR1 and mouse Mdr1a proteins were expressed in the brain capillaries of the hMDR1 mice although mouse Mdr1a should be completely replaced with human MDR1 ideally. The protein expression levels of human MDR1 and mouse Mdr1a in the brain capillaries of the hMDR1 mice were 0.355 and 2.45 fmol/μg protein, respectively, which were 63- and 9.1-fold smaller than that of Mdr1a in C57BL/6 WT mice, respectively. These results suggest that the lower extent of the drug efflux at the BBB of the hMDR1 mice was caused by the lower expression of P-gp proteins including human MDR1 and mouse Mdr1a than that in the C57BL/6 WT mice. Further development and refinement of this model is required to produce a reliable model with sufficient expression levels of human P-gp and without the protein expression of mouse Mdr1a to study the species differences in the functionality of P-gp at the BBB. Therefore, functional validation combined with QTAP analysis is valuable tool to validate a variety of newly generated humanized mouse models.

The Mdr1a/b(-/-) mice are used as a standard model for investigating the role of P-gp in drug development [24–26]. These mice were used in the present study as a reference with which to compare the importance of P-gp for the BBB transport of the selected compounds. The 20, 30 and 4 times higher K_p_ for digoxin, verapamil and docetaxel in Mdr1a/1b(-/-) mice as compared to the FVB WT controls confirmed the major role of P-gp in their efflux from the brain, and are in agreement with previous studies [27–29]. For verapamil, lower expression levels of P-gp in hMDR1 mice explains the similar Kp,brain values in hMDR1 and Mdr1a/1b(-/-) mice. For digoxin, this low expression of P-gp seems to be efficient enough to keep the Kp,brain increase to only 2-fold, in comparison to 20-fold increase in Mdr1a/1b(-/-) mice. As digoxin is a low permeable compound, while verapamil is highly permeable, the difference observed may be explained by this fact i.e. that less P-gp is needed to efflux digoxin than verapamil. Interestingly, slight but statistically significant differences in digoxin and verapamil brain K_p_ values were observed even between the FVB and C57BL/6 WT mice, indicating certain differences in P-gp function and/or expression between the two mouse lines.

The K_p, uu_ is a pharmacologically relevant descriptor for the extent of brain distribution of a drug that show the relative importance of efflux or influx at the BBB [30]. Digoxin, verapamil, docetaxel and oxycodone all had a trend of higher K_p, uu_ in hMDR1 mice as compared to the C57BL/6 WT controls (Table 1). In the C57BL/6 WT and hMDR1 mice the value of K_p, uu_ for oxycodone was calculated to be 1.9 and 3.0 and for verapamil it was 0.1 and 1.6, respectively. The oxycodone result is very close to the previous studies in rats, where oxycodone had an active uptake at the BBB with K_p, uu_ of 3 in rats [31, 32]. Oxymorphone, a metabolite of oxycodone also exhibit active uptake transport at the BBB [33]. The information for verapamil is however new, indicating that this strong P-gp substrate actually also has an active uptake at the BBB, as shown by the K_p, uu_ for verapamil being above unity in the hMDR1 mice. Active uptake of verapamil was reported with a cation transporter at the blood-retinal barrier into human and rat retinal pigment epithelial cells [17, 22].

We also studied the effect of different blockers (i.e., cyclosporine A, verapamil, elacridar and quinidine) on the transport of digoxin in hMDR1 and WT mice. All blockers resulted in a larger increase in digoxin K_p_ in the C57BL/6 WT mice compared to in the hMDR1 mice.

The oxycodone K_p_ was not changed after co-administration of the rather specific P-gp blocker elacridar in comparison to the control mice that were administered oxycodone only. This result is in agreement with previous results in rats where administration of the P-gp blocker PSC833 was found to have no effect on the tail-flick latency of oxycodone [34].

Comparisons of expression levels of hMDR1, Mdr1a and Mdr1b at the BBB between hMDR1 and C57BL/6 WT mice are important to understand the mechanisms of differences in BBB penetration of P-gp substrates between these mice. Protein levels reflect the activities of transporters better than mRNA levels. Antibody-based analysis cannot distinguish human MDR1, mouse Mdr1a and Mdr1b and also lacks the accuracy of quantification, but QTAP analysis enables us to quantify the protein expressions of these three proteins separately with high quantitative performance. Therefore, we applied the QTAP analysis for the evaluation of hMDR1 mice in the present study.

The QTAP analysis provided clear reasons why the efflux activity of P-gp in hMDR1 mice was smaller than that in the C57BL/6 WT mice; it could be because of lower protein expression levels of human MDR1 and the presence of remaining mouse Mdr1a at the BBB in the hMDR1 mice. Although it is unclear whether the Mdr1a proteins detected in hMDR1 mice are functional or not, the efflux activity of P-gp observed in the hMDR1 mice could be due to the remaining protein expression of mouse Mdr1a, because hMDR1 levels were almost negligible. The QTAP analysis provided us with these clear understandings for the reason of difference in BBB penetration of P-gp substrates by the separate quantification of hMDR1, Mdr1a and Mdr1b proteins. This cannot be achieved by antibody-based analyses as there is no specific antibody to distinguish these three subtypes. For not only P-gp, but also for a variety of other functional molecules, it is necessary to distinguish human and mouse homologs in the validation of humanized mice. Although PCR analysis can distinguish them, the mRNA expression levels do not always reflect the protein expression levels which are the final productions of functional molecules [1]. From these points of view, QTAP is a useful methodology for the evaluation of expression of target molecules in humanized mice.

## Conclusion

The present study evaluated the hMDR1 mouse model that the efflux activities of P-gp at the BBB were lower than those in the wild-type mice because of lower protein expression levels of total P-gp including the human MDR1 and the unexpectedly remaining mouse Mdr1a in the brain capillaries. Although the present hMDR1 mice have been determined to be insufficient humanized mice, the combination of in vivo and QTAP analyses successfully assessed the deficiency of the hMDR1 mice. Therefore, this approach is an important tool in future to validate the humanized animal models.

## Supporting Information

S1 Table Peptide probe sequences and selected ions for quantification of mouse mdr1a and mdr1b with LC-MS/MS. (DOCX)

## References

[1] Uchida Y, Ohtsuki S, Katsukura Y, Ikeda C, Suzuki T, Kamiie J, et al Quantitative targeted absolute proteomics of human blood-brain barrier transporters and receptors. Journal of neurochemistry. 2011;117(2):333–45. 10.1111/j.1471-4159.2011.07208.x 21291474

[2] Tsuji A, Terasaki T, Takabatake Y, Tenda Y, Tamai I, Yamashima T, et al P-glycoprotein as the drug efflux pump in primary cultured bovine brain capillary endothelial cells. Life sciences. 1992;51(18):1427–37. 1357522 10.1016/0024-3205(92)90537-y

[3] Juliano RL, Ling V. A surface glycoprotein modulating drug permeability in Chinese hamster ovary cell mutants. Biochimica et biophysica acta. 1976;455(1):152–62. 990323 10.1016/0005-2736(76)90160-7

[4] Schinkel AH. P-Glycoprotein, a gatekeeper in the blood-brain barrier. Advanced drug delivery reviews. 1999;36(2–3):179–94. 10837715 10.1016/s0169-409x(98)00085-4

[5] Katoh M, Suzuyama N, Takeuchi T, Yoshitomi S, Asahi S, Yokoi T. Kinetic analyses for species differences in P-glycoprotein-mediated drug transport. Journal of pharmaceutical sciences. 2006;95(12):2673–83. 16892207 10.1002/jps.20686

[6] Syvanen S, Lindhe O, Palner M, Kornum BR, Rahman O, Langstrom B, et al Species differences in blood-brain barrier transport of three positron emission tomography radioligands with emphasis on P-glycoprotein transport. Drug metabolism and disposition: the biological fate of chemicals. 2009;37(3):635–43. 19047468 10.1124/dmd.108.024745

[7] Gottesman MM, Pastan I. Biochemistry of multidrug resistance mediated by the multidrug transporter. Annual review of biochemistry. 1993;62:385–427. 8102521 10.1146/annurev.bi.62.070193.002125

[8] Hsu SI, Lothstein L, Horwitz SB. Differential overexpression of three mdr gene family members in multidrug-resistant J774.2 mouse cells. Evidence that distinct P-glycoprotein precursors are encoded by unique mdr genes. The Journal of biological chemistry. 1989;264(20):12053–62. 2473069

[9] Schinkel AH, Smit JJ, van Tellingen O, Beijnen JH, Wagenaar E, van Deemter L, et al Disruption of the mouse mdr1a P-glycoprotein gene leads to a deficiency in the blood-brain barrier and to increased sensitivity to drugs. Cell. 1994;77(4):491–502. 7910522 10.1016/0092-8674(94)90212-7

[10] Sasongko L, Link JM, Muzi M, Mankoff DA, Yang X, Collier AC, et al Imaging P-glycoprotein transport activity at the human blood-brain barrier with positron emission tomography. Clinical pharmacology and therapeutics. 2005;77(6):503–14. 15961982 10.1016/j.clpt.2005.01.022

[11] Cutler L, Howes C, Deeks NJ, Buck TL, Jeffrey P. Development of a P-glycoprotein knockout model in rodents to define species differences in its functional effect at the blood-brain barrier. Journal of pharmaceutical sciences. 2006;95(9):1944–53. 16850390 10.1002/jps.20658

[12] Hogan BLM, Beddington RSP, Costantini F, Lacy E. Manipulating the Mouse Embryo: A Laboratory Manual. 2nd ed ed. Plainview, New York: Cold Spring Harbor Lab. Press; 1994 p. 253–89. 10.1007/978-1-60327-019-9_13

[13] Scheer N, Ross J, Rode A, Zevnik B, Niehaves S, Faust N, et al A novel panel of mouse models to evaluate the role of human pregnane X receptor and constitutive androstane receptor in drug response. J Clin Invest. 2008;118(9):3228–39. 10.1172/JCI35483 18677425 PMC2493444

[14] Friden M, Gupta A, Antonsson M, Bredberg U, Hammarlund-Udenaes M. In vitro methods for estimating unbound drug concentrations in the brain interstitial and intracellular fluids. Drug metabolism and disposition: the biological fate of chemicals. 2007;35(9):1711–9. 17591680 10.1124/dmd.107.015222

[15] Banker MJ, Clark TH, Williams JA. Development and validation of a 96-well equilibrium dialysis apparatus for measuring plasma protein binding. Journal of pharmaceutical sciences. 2003;92(5):967–74. 12712416 10.1002/jps.10332

[16] Friden M, Ducrozet F, Middleton B, Antonsson M, Bredberg U, Hammarlund-Udenaes M. Development of a high-throughput brain slice method for studying drug distribution in the central nervous system. Drug metabolism and disposition: the biological fate of chemicals. 2009;37(6):1226–33. 19299522 10.1124/dmd.108.026377

[17] Loryan I, Friden M, Hammarlund-Udenaes M. The brain slice method for studying drug distribution in the CNS. Fluids and barriers of the CNS. 2013;10(1):6 10.1186/2045-8118-10-6 23336814 PMC3602653

[18] Keilhoff G, Wolf G. Comparison of double fluorescence staining and LDH-test for monitoring cell viability in vitro. NeuroReport. 1993;5(2):129–32. 7906556 10.1097/00001756-199311180-00008

[19] Choksakulnimitr S, Masuda S, Tokuda H, Takakura Y, Hashida M. In vitro cytotoxicity of macromolecules in different cell culture systems. Journal of Controlled Release. 1995;34(3):233–41.

[20] Friden M, Ljungqvist H, Middleton B, Bredberg U, Hammarlund-Udenaes M. Improved measurement of drug exposure in the brain using drug-specific correction for residual blood. Journal of cerebral blood flow and metabolism: official journal of the International Society of Cerebral Blood Flow and Metabolism. 2010;30(1):150–61. 10.1038/jcbfm.2009.200 PMC2949109 19756019

[21] Friden M, Bergstrom F, Wan H, Rehngren M, Ahlin G, Hammarlund-Udenaes M, et al Measurement of unbound drug exposure in brain: modeling of pH partitioning explains diverging results between the brain slice and brain homogenate methods. Drug metabolism and disposition: the biological fate of chemicals. 2011;39(3):353–62. 21149540 10.1124/dmd.110.035998

[22] Kamiie J, Ohtsuki S, Iwase R, Ohmine K, Katsukura Y, Yanai K, et al Quantitative atlas of membrane transporter proteins: development and application of a highly sensitive simultaneous LC/MS/MS method combined with novel in-silico peptide selection criteria. Pharmaceutical research. 2008;25(6):1469–83. 10.1007/s11095-008-9532-4 18219561

[23] Agarwal S, Uchida Y, Mittapalli RK, Sane R, Terasaki T, Elmquist WF. Quantitative proteomics of transporter expression in brain capillary endothelial cells isolated from P-glycoprotein (P-gp), breast cancer resistance protein (Bcrp), and P-gp/Bcrp knockout mice. Drug metabolism and disposition: the biological fate of chemicals. 2012;40(6):1164–9. 22401960 10.1124/dmd.112.044719 PMC3362790

[24] Schinkel AH. Pharmacological insights from P-glycoprotein knockout mice. International journal of clinical pharmacology and therapeutics. 1998;36(1):9–13. 9476142

[25] Chen C, Liu X, Smith BJ. Utility of Mdr1-gene deficient mice in assessing the impact of P-glycoprotein on pharmacokinetics and pharmacodynamics in drug discovery and development. Current drug metabolism. 2003;4(4):272–91. 12871045 10.2174/1389200033489415

[26] Lagas JS, Vlaming ML, Schinkel AH. Pharmacokinetic assessment of multiple ATP-binding cassette transporters: the power of combination knockout mice. Molecular interventions. 2009;9(3):136–45. 10.1124/mi.9.3.7 19592674

[27] Schinkel AH, Wagenaar E, van Deemter L, Mol CA, Borst P. Absence of the mdr1a P-Glycoprotein in mice affects tissue distribution and pharmacokinetics of dexamethasone, digoxin, and cyclosporin A. J Clin Invest. 1995;96(4):1698–705. 7560060 10.1172/JCI118214 PMC185805

[28] Hendrikse NH, Schinkel AH, de Vries EG, Fluks E, Van der Graaf WT, Willemsen AT, et al Complete in vivo reversal of P-glycoprotein pump function in the blood-brain barrier visualized with positron emission tomography. British journal of pharmacology. 1998;124(7):1413–8. 9723952 10.1038/sj.bjp.0701979 PMC1565536

[29] Kemper EM, Verheij M, Boogerd W, Beijnen JH, van Tellingen O. Improved penetration of docetaxel into the brain by co-administration of inhibitors of P-glycoprotein. European journal of cancer (Oxford, England: 1990). 2004;40(8):1269–74. 15110893 10.1016/j.ejca.2004.01.024

[30] Hammarlund-Udenaes M, Friden M, Syvanen S, Gupta A. On the rate and extent of drug delivery to the brain. Pharmaceutical research. 2008;25(8):1737–50. 18058202 10.1007/s11095-007-9502-2 PMC2469271

[31] Bostrom E, Simonsson US, Hammarlund-Udenaes M. In vivo blood-brain barrier transport of oxycodone in the rat: indications for active influx and implications for pharmacokinetics/pharmacodynamics. Drug metabolism and disposition: the biological fate of chemicals. 2006;34(9):1624–31. 16763013 10.1124/dmd.106.009746

[32] Sadiq MW, Borgs A, Okura T, Shimomura K, Kato S, Deguchi Y, et al Diphenhydramine active uptake at the blood-brain barrier and its interaction with oxycodone in vitro and in vivo. Journal of pharmaceutical sciences. 2011;100(9):3912–23. 10.1002/jps.22567 21472729

[33] Sadiq MW, Bostrom E, Keizer R, Bjorkman S, Hammarlund-Udenaes M. Oxymorphone active uptake at the blood-brain barrier and population modeling of its pharmacokinetic-pharmacodynamic relationship. Journal of pharmaceutical sciences. 2013;102(9):3320–31. 10.1002/jps.23492 23463542

[34] Bostrom E, Simonsson US, Hammarlund-Udenaes M. Oxycodone pharmacokinetics and pharmacodynamics in the rat in the presence of the P-glycoprotein inhibitor PSC833. Journal of pharmaceutical sciences. 2005;94(5):1060–6. 15799017 10.1002/jps.20327

