# Supplementary material for: Validation of a P-Glycoprotein (P-gp) Humanized Mouse Model by Integrating Selective Absolute Quantification of Human MDR1, Mouse Mdr1a and Mdr1b Protein Expressions with In Vivo Functional Analysis for Blood-Brain Barrier Transport
Source: PLoS One. 2015 May 1;10(5):e0118638. doi: 10.1371/journal.pone.0118638 (PMC4416786; doi:10.1371/journal.pone.0118638)
Supplement: S1 Table — (DOCX) [file pone.0118638.s001.docx]

| **Synonym (Gene name)** | **Probe Sequence** | **Peptide Mass** | **SRM/MRM transitions** | | | | |
| --- | --- | --- | --- | --- | --- | --- | --- |
|  |  |  | **Q1** | **Q3-1** | **Q3-2** | **Q3-3** | **Q3-4** |
| Mouse mdr1a | VVSYEEIVR | 1092.6 | 547.3 | 895.5 | 645.4 | 808.4 | 516.3 |
| *(Abcb1a)* | VVSYEEIV**R*** | 1102.6 | 552.3 | 905.5 | 655.4 | 818.4 | 526.3 |
| Mouse mdr1b | TVIAFGGQQK | 1047.6 | 524.8 | 848.5 | 664.3 | 735.4 | 517.3 |
| *(Abcb1b)* | TVIAFGGQQ**K*** | 1055.6 | 528.8 | 856.5 | 672.3 | 743.4 | 525.3 |

The selected peptides were synthesized and their purity was checked with HPLC-UV according to Kamiie et al., *Pharm Res* 2008. The SRM/MRM transitions were determined from MS/MS spectra obtained by direct infusion of 100 nM peptide solution at a flow rate of 5 µL/min with a syringe pump (Harvard) into the mass spectrometer. Doubly charged precursor ions were selected (Q1). Four transitions per peptide (Q3-1, -2, -3 and -4), corresponding to high-intensity fragment ions, were selected. The declustering potentials and collision energies were optimized to maximize signal strength. For the stable isotope-labeled peptides, precursor ions and transitions corresponding to those of the unlabeled peptides were selected, with the same declustering potentials and collision energies as for the unlabeled peptides. Bold letters with asterisks indicate amino acid residues labeled with stable isotope (^13^C and ^15^N).
